# Global trends in research on MOG antibody-associated disease: bibliometrics and visualization analysis

**DOI:** 10.3389/fimmu.2024.1278867

**Published:** 2024-02-02

**Authors:** Shuhan Zheng, Yang Wang, Jiaming Geng, Xueyan Liu, Liang Huo

**Affiliations:** ^1^ Department of Pediatrics, Shengjing Hospital of China Medical University, Shenyang, China; ^2^ National Science Library, Chinese Academy of Sciences, Beijing, China; ^3^ Department of Information Resources Management, School of Economics and Management, University of Chinese Academy of Sciences, Beijing, China; ^4^ Department of Pharmaceutical Biotechnology, China Medical University-The Queen’s University if Belfast Joint College, Shenyang, China

**Keywords:** global trends, MOG antibody associated disease, bibliometric, visualization analysis, VOSviewer, Citespace

## Abstract

**Objective:**

The purpose of this study was to investigate the current research status, focus areas, and developmental trends in the field of Myelin oligodendrocyte glycoprotein antibody-associated disease (MOGAD) through an analysis of scientific literature.

**Methods:**

The relevant research articles on MOGAD published from 1947 to 2022 were retrieved from the Web of Science database. The quantitative output of MOGAD related research articles, their distribution by country/region, data on collaborative publishing, influential authors, high-yield institutions, keywords, hotspots, and development trends were analyzed. Additionally, visual knowledge maps were generated using VOSviewer and Citespace.

**Results:**

There has been a steady increase in the number of MOGAD related publications indicating that the subject has garnered increasing interest among researchers globally. The United States has been the leading contributor with 496 papers (19.25%), followed by China (244, 9.63%), Japan (183, 7.10%), the United Kingdom (154, 5.98%), and Germany (149, 5.78%). Among these countries, the United Kingdom boasts the highest citation frequency at the rate of 46.49 times per paper. Furthermore, active collaboration in MOGAD related research is observed primarily between the United States and countries such as Canada, Germany, Australia, Italy, the United Kingdom and Japan. Mayo Clinic ranks first in total articles published (109) and frequency of citations per article (77.79). Takahashi Toshiyuki from Tohoku University is the most prolific author, while *Multiple Sclerosis and Related Disorders* is the most widely read journal in this field. “Disease Phenotype”, “Treatment”, “Novel Coronavirus Infection and Vaccination”, “Immunopathological Mechanisms”, “Clinical characteristics of children” and “Prognosis” are the primary keywords clusters in this field. “Novel Coronavirus Infection and Vaccination” and “Immunopathological Mechanisms” are research hotspots and have great development potential.

**Conclusion:**

The past three decades have witnessed a significant expansion of research on MOGAD. The pathogenetic mechanism of MOGAD is poised to be the prominent research focus in this field in the foreseeable future.

## 1 Introduction

Myelin oligodendrocyte glycoprotein antibody-associated disease (MOGAD) is a newly-defined demyelinating disorder of the central nervous system (CNS) with antibodies against myelin oligodendrocyte glycoprotein (MOG) that are expressed on the surface of oligodendrocytes and myelin sheath in the central nervous system (1–6). The clinical phenotype of this demyelinating disease is distinct from that of aquaporin-4 (AQP4) IgG-positive neuromyelitis optica Spectrum disorders (NMOSD) and multiple sclerosis (MS) (7–9) The pathogenesis of MOGAD is believed to be related to the demyelination process mediated by antibody/complement, but the specific pathogeneticmechanism is yet to be elucidated (3, 10). The epidemiological data of MOGAD based on geographic region and ethnicity is limited. The female-to-male ratio is about 1:1, and the estimated annual incidence is 1.6 per million person-years. There are differences between children and adults regarding the incidence, clinical phenotype, treatment, recurrence, and prognosis (7, 11–13).The most common clinical manifestations of MOGAD are optic neuritis (ON), myelitis, and acute disseminated encephalomyelitis (ADEM) which most frequently occurs in children (12, 14). The severity of MOGAD varies, with about 3% of severe patients requiring mechanical ventilation support during the acute phase of the disease (15).Approximately 40%-50% of M (16)OGAD patients maintain a monophasic course while 50-60% of cases experience disease relapses (17–19).The risk of recurrence may be related to the persistence of MOG-IgG seropositivity after the first attack. Moreover, the utility of routine magnetic resonance imaging (MRI) to monitor disease activity was limited by the course of MOGAD with no disease activity between episodes (16, 20, 21). High-dose corticosteroids are effective in treating MOGAD, but early treatment with intravenous gamma globulin or plasmapheresis is recommended for patients with severe disease or high potential for disability (5, 22). Most patients with MOGAD have a good prognosis (23–25).The possibility of legacy nervous system dysfunction is significantly higher in children (11, 24, 26).The international consensus diagnostic criteria for MOGAD are currently being developed. Due to the lack of prospective randomized controlled trials, there are many unresolved questions about the pathogenesis, clinical features, and treatment of MOGAD.

Bibliometrics is an important approach for quantitative measurement of research in a particular field. It entails the use of statistical methods for analysis of factors such as the temporal trend of technological development, future trends, and global scientific and technological competitiveness in a specific field based on published data including papers and patents (27). In recent years, bibliometric analysis has been widely adopted for analysis of massive scientific research data and identification of developing trends (28). Bibliometric analysis methods in this study are necessary and valuable. First, bibliometric analysis method can analyze the current situation and development trend of the discipline from an objective perspective, without individual subjective judgment. So, the data results are more objective. Secondly, compared to the other disciplines, researchers in the medical field tend to choose journal papers as the form of publication. So medical paper data can better reflect the current development status, research hotspots, and future trends. It is necessary and appropriate to use medical data as the research object of the discipline trend. Finally, some researchers in the medical field have used bibliometric methods for review and analysis, and the research results have also been recognized by peers (29–36). Therefore, bibliometric methods to study MOGAD is feasible. To the best of our knowledge, this is the first study that applied bibliometric visualization methods into systematic investigation about the year of publication, countries/regions, organizations, active authors, journals, references, keywords and hotspots involved in MOGAD research. We combined the quantitative and qualitative comprehensive analysis to evaluate the progress and evolution of MOGAD by using the VOSViewer and Citespace software. We considered that this study performed visualization analysis from 1947 to 2022 to identify cooperation networks, track research trends and highlight current hotspots. It can not only help researchers identify its significant features and predict future research directions, but also provide them with meaningful guidance in the selection of frontier topics.

## 2 Data and methods

### 2.1 Data acquisition

**Figure 1:**
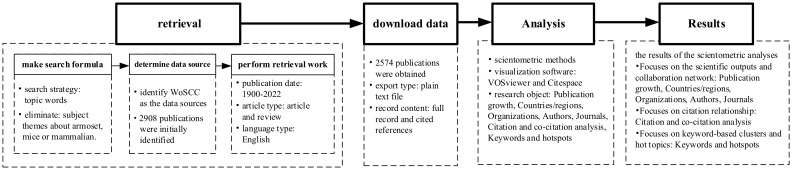
Research process applied in this study.

The data in this study were obtained from Web of Science core collection (WoSCC) by Clarivate Analytics. This database is the earliest citation index database in the world and contains>150million highly recognized and authoritative social science and science citations. The database offers full functionality for literature search, citation analysis, and data export to facilitate data processing. In this study, keywords related to MOGAD were selected with the search formula: TS=((“MOG antibody associated” or “MOG associated” or “Myelin Oligodendrocyte Glycoprotein antibody associated” or “Myelin Oligodendrocyte Glycoprotein associated” or “MOG INDUCE*” or “Myelin Oligodendrocyte Glycoprotein INDUCE*” or “MOG antibody” or “Myelin Oligodendrocyte Glycoprotein” or myelitis or medullitis or acutemyelitis or rachiomyelitis or rhachiomyelitis or “optic neuromyelitis” or “neuromyelitisoptica” or “ophthalmoneuromyelitis” or “neurooptic myelitis” or “neuromyelitis optical” or “Optic neuritis” or neuropapillitis or “neuritis optica” or “optic neuvitis” or “acute disseminated encephalomyelitis” or “multiphasic acute disseminated encephalomyelitis” or “ADEM” or “multiphasic ADEM”) and (demyelinat* or myelinolysis)) not TS=(marmoset* or mice or mouse or rat or murine or animal* or macaque* or mammalian). The reference period for the literature search was from 1900 to 2022. The language type was set to English, and the article type was set to article and review. After removing articles unrelated to MOGAD, 2574 publications were obtained. The export type of publication was plain text file and the content included full record and cited references. The retrieval framework is shown in **Figure 1**.

### 2.2 Methods

Bibliometrics refers to the study of bodies of interrelated documents. It is a methodology for the analysis and visualization of the connections among research topics, researchers, affiliations, or journals. In this study, WOS-based literature analysis was conducted to understand the distribution of year of publication, journals, organizations, authors, and research fields. The Standard Competition Ranking method was used to determine the ranking order. Subsequently, the VOSviewer and Citespace software tool were used to map collaborations (co-authorships), topics (term co-occurrence), and citation patterns (bibliographic coupling). The results of the scientometric analyses focuse on 3 themes, which is the scientific outputs and collaboration network, the citation relationship and keyword-based clusters and hot topics. Specifically, the sections of Publication growth, Countries/regions, Organizations, Authors, Journals focuse on the scientific outputs and collaboration network. The section of Citation and co-citation analysis focuses on citation relationship, while the section of Keywords and hotspots focuses on keyword-based clusters and hot topics.

## 3 Results

### 3.1 Publication growth

**Figure 2:**
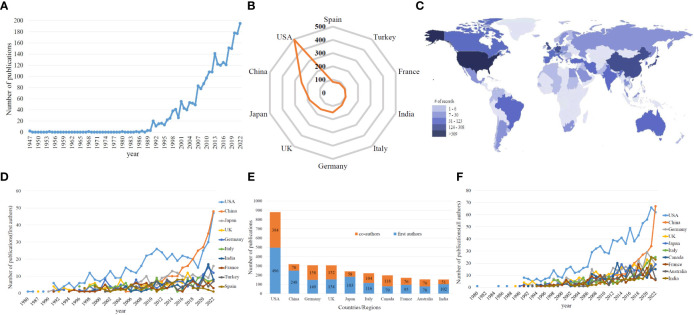
**(A)** The global number of publications related to MOG research. **(B)** The sum of publications related to MOGAD research from the top 10 countries/regions based on the first authors. **(C)** Distribution of MOGAD research in world map based on first authors. **(D)** The annual number of publications in the top 10 most productive countries based on the first authors from 1980 to 2022. **(E)** The sum of publications related to MOGAD research from the top 10 countries/regions based on the first and co-authors. **(F)** The annual number of publications in the top 10 most productive countries based on the first and co-authors from 1980 to 2022.

The chronological distribution of published articles is shown in **Figure 2A**. The data showed a steady increase in the publications related to MOGAD. Research articles related to MOGAD were published as early as the 1940s. However, before the 1990s, the annual output of relevant research articles was less than 5. Since the 1990s, there was a rapid increase in the output of MOGAD research articles to over 10 articles per year. In 2011, the number exceeded 100 for the first time, reaching 108, which shows a rapid upward trend. In the past few years, there has been a gradual increase in the number of published studies, peaking at 171 in 2021.Theincreasing trend in the number of published articles suggests that MOGAD will continue to be a research hotspot in the foreseeable future.

**Figure 2B** shows the country/region statistics of the institution where the first author is located. Seventy-two countries/regions have contributed to the literature in this field. The output of top 10 countries accounts for nearly 70% of the total number of papers. The United States (USA) has published the most papers (496, 19.25%), followed by China (244, 9.63%), Japan (183, 7.10%), the United Kingdom (UK) (154, 5.98%) and Germany (149, 5.78%). **Figure 2C** shows that East Asia, North America, and West Europe were the regions making the top contributions to the number of publications. As shown in **Figure 2D**, the number of papers published annually by the top 10 countries/regions increased from 1 in 1980 to 158 in 2022, accounting for 81.03% of the total global publications in 2022. The United States ranked first with 496 articles, far exceeding other countries/regions in terms of quantity. China and Japan ranked second and third with 244 and 183 articles published, respectively. These results indicate that MOGAD has attracted increasing attention from researchers and has reached a stage of rapid development. Statistical analysis of the first author country can demonstrate the dominant position of MOGAD, while statistical analysis of each author country can reflect the international cooperation status. As shown in **Figure 2E**, the number of articles participated by American researchers is the highest, with a total of 880 articles, followed by 318 articles from China and 307 articles from Germany. The proportion of co-authors articles in most countries/regions is around 50%. Especially in the United States, the number of co-authors articles has grown rapidly since 2012, and co-authors from other countries led to USA consistently maintaining the highest number of articles every year (**Figure 2F**).

### 3.2 Countries/Regions

**Figure 3:**
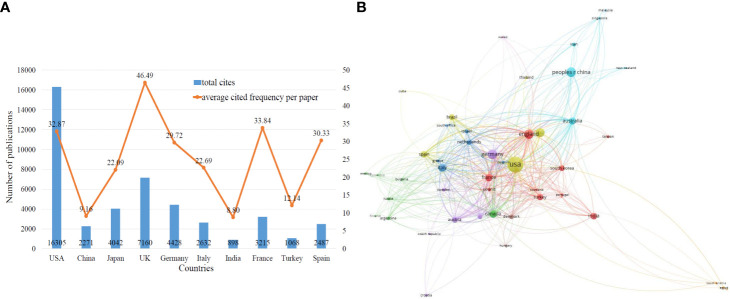
**(A)** The total cites (blue bars) and average cited frequency per paper (red curve) of publications related to MOGAD research from the top 10 countries. **(B)** Cooperation network of country/regions.

The frequency of citations can reflect the quality of the paper to a certain extent. The higher the frequency of citations, the greater is the attention and recognition of researchers in the same field. **Figure 3A** shows the top 10 countries ranked according to the frequency of citations. On comparing the number of papers, total citation frequency, and average citation frequency, it can be seen that the USA, the UK, and Germany rank among the top three in terms of total citation frequency. The UK has a higher citation frequency, reaching 46.49 times per paper. China ranks second in terms of output of MOGAD-related research papers, but the citation frequency of Chinese publications is 9.16 times per article.

The cooperation network of the publications can visually display the status of scientific and technological cooperation among countries/regions. In this network map, two countries/regions collaborating to publish papers are connected by line segments. The thicker the line segments, the closer is the degree of cooperation and the colors are the same. As shown in **Figure 3B**, USA has the most active cooperation in the field of MOGAD, with frequent cooperation with countries such as Canada, Germany, Australia, Italy, the UK, and Japan. In addition, Australia has shown close cooperation with Germany. China has also actively participated in international cooperation, with most notable cooperation with Australia, the USA, the UK, Canada, Germany, France, and Italy.

### 3.3 Organizations

**Table 1** shows the status of the institutions in the field of MOGAD research. United States accounts for the largest share among the top 10 institutions. Mayo Clinic ranks first with a total of 109 articles published, and the frequency of citations per article is as high as 77.79. Mayo Clinic is a nonprofit organization committed to clinical practice, education, and research with a history of over a hundred years. It is now the leading institution in the world in terms of MOGAD related research articles. University College London ranked second with 85 articles. In addition, Medical University of Vienna in Austria has published 49 articles about MOGAD, and the frequency of citations per article is as high as 81, which to some extent reflects the high quality of its articles.

**Table 1:** Top 10 institutions in the field of MOGAD related research.

Rank	Institution Name	Publication counts	Cited frequency (times)	Citation frequency per article (times/article)
1	Mayo Clinic	109	8479	77.79
2	University College London	85	6484	76.28
3	University of Sydney	64	2846	44.47
4	Tohoku University	58	2744	47.31
5	Harvard University	51	1862	36.51
6	University of California	51	2591	50.80
7	University of Toronto	51	2259	44.29
8	University of Pennsylvania	50	2135	42.70
9	Medical University of Innsbruck	49	3044	62.12
10	Medical University of Vienna	49	3969	81.00

**Figure 4:**
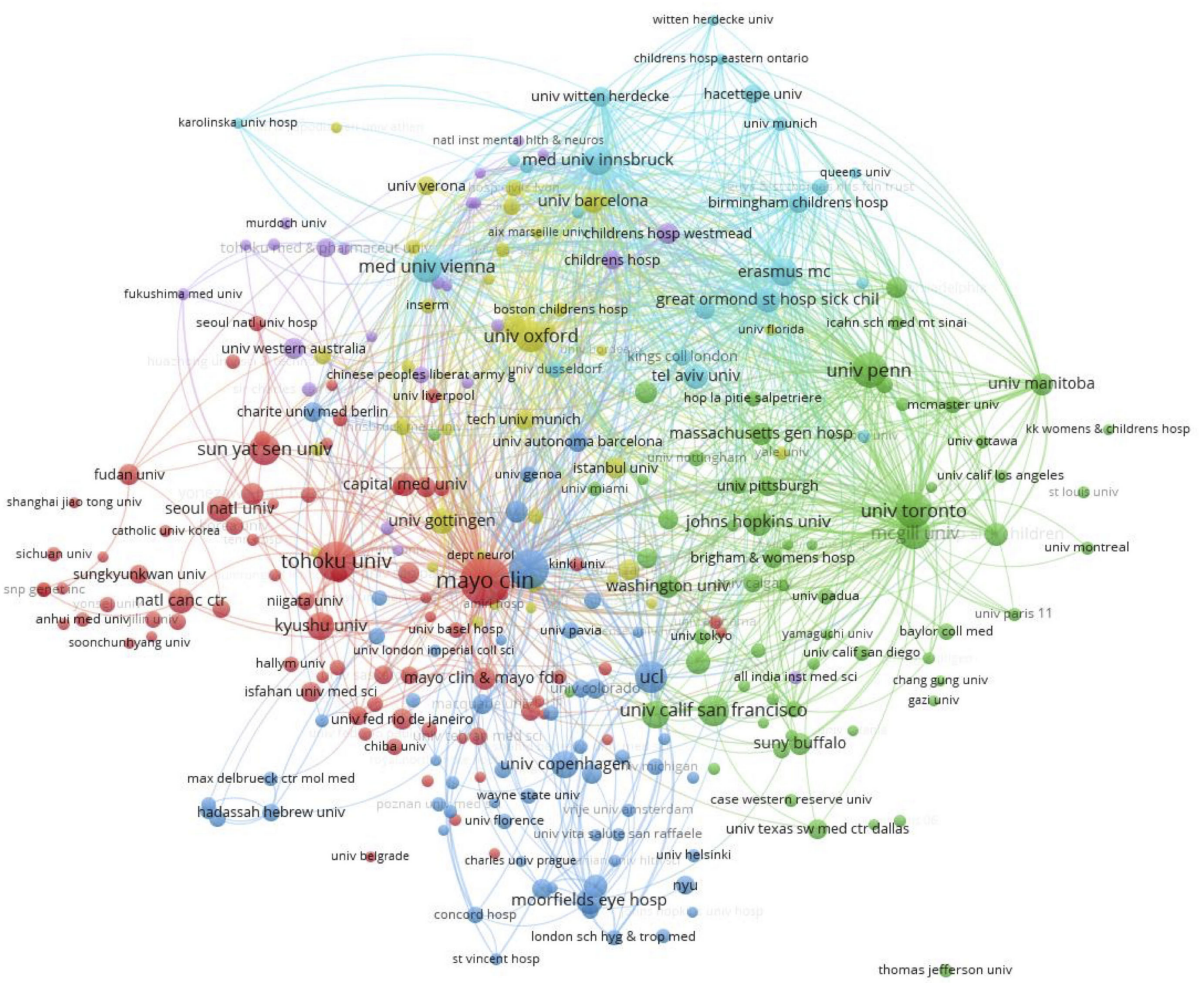
Cooperation network of institutions.

**Figure 4** shows the cooperative network relationships of the institutions. It shows frequent collaboration between institutions from different countries/regions, such as Mayo Clinic in the USA, Medical University of Vienna in Austria (correlation degree=0.50), and University of Gottingen in Germany (correlation degree=0.60); Spain’s Autonomous University of collaborates closely with the UK’s UCL (correlation degree=0.58) and Austria’s Medical University of Innsbruck (correlation degree=0.83). University of California, USA collaborates more frequently with Johns Hopkins University (correlation degree=0.82), University of Oxford and Great Ormond Street Hospital in the UK (correlation degree=0.79), and University of British Columbia and University of Toronto in Canada (correlation degree=0.68) with strong connections.

### 3.4 Authors

In order to demonstrate the influence of important authors comprehensively, the indicators of the publication counts and citation weight are chosen to represent. **Table 2** shows the influential authors of publication counts and citations weight. Takahashi Toshiyuki from Tohoku University has the highest number of publications (37 publications). The second ranked researchers are Reindl Markus from Medical University of Innsbruck, Banwell Brenda from University of Pennsylvania, and Pittock Sean J from Mayo Clinic, all of whom have published 31 papers each. Dale Russell C of University of Sydney has published 29 papers, ranking third. The citations weights of Lucchinetti, Claudia F (citations weight=110.39) and Pittock, Sean J (citations weight=102.90) from Mayo Clin are over 100. And the citation weight ranks of Lassmann, Hans (citations weight=94.49), Vincent, Angela (citations weight=67.94), Flanagan, Eoin P. (citations weight=67.54), Jacqueline, Palace (citations weight=62.00), Jacob, Anu (citations weight=58.01) are in the TOP10.

**Table 2:** Influential authors in the field of MOGAD related research.

Rank	Authors	Institutions	Publication counts	Rank	Authors	Institutions	Citations weight
1	Takahashi, Toshiyuki	Tohoku Univ	37	1	Lucchinetti, Claudia F	Mayo Clin	110.39
2	Reindl, Markus	Innsbruck Med Univ	31	2	Pittock, Sean J	Mayo Clin	102.90
2	Banwell, Brenda	Univ Penn	31	3	Lassmann, Hans	Medical University of Vienna	94.75
2	Pittock, Sean J	Mayo Clin	31	4	Waters, Patrick	Univ Oxford	94.49
5	Dale, Russell C	Univ Sydney	29	5	Weinshenker, Brian G	Innsbruck Med Univ	80.16
6	Lucchinetti, Claudia F	Mayo Clin	28	6	Vincent, Angela	Univ Oxford	67.94
7	Fujihara, Kazuo	Tohoku Univ	26	7	Flanagan, Eoin P.	Mayo Clin	67.54
8	Nakashima, Ichiro	Tohoku Univ	25	8	Reindl, Markus	Innsbruck Med Univ	66.85
9	Waters, Patrick	Univ Oxford	23	9	Jacqueline, Palace	Univ Oxford	62.00
9	Brilot, Fabienne	Univ Sydney	23	10	Jacob, Anu	Walton Ctr NHS Trust	58.01
9	Weinshenker, Brian G	Innsbruck Med Univ	23				

**Figure 5:**
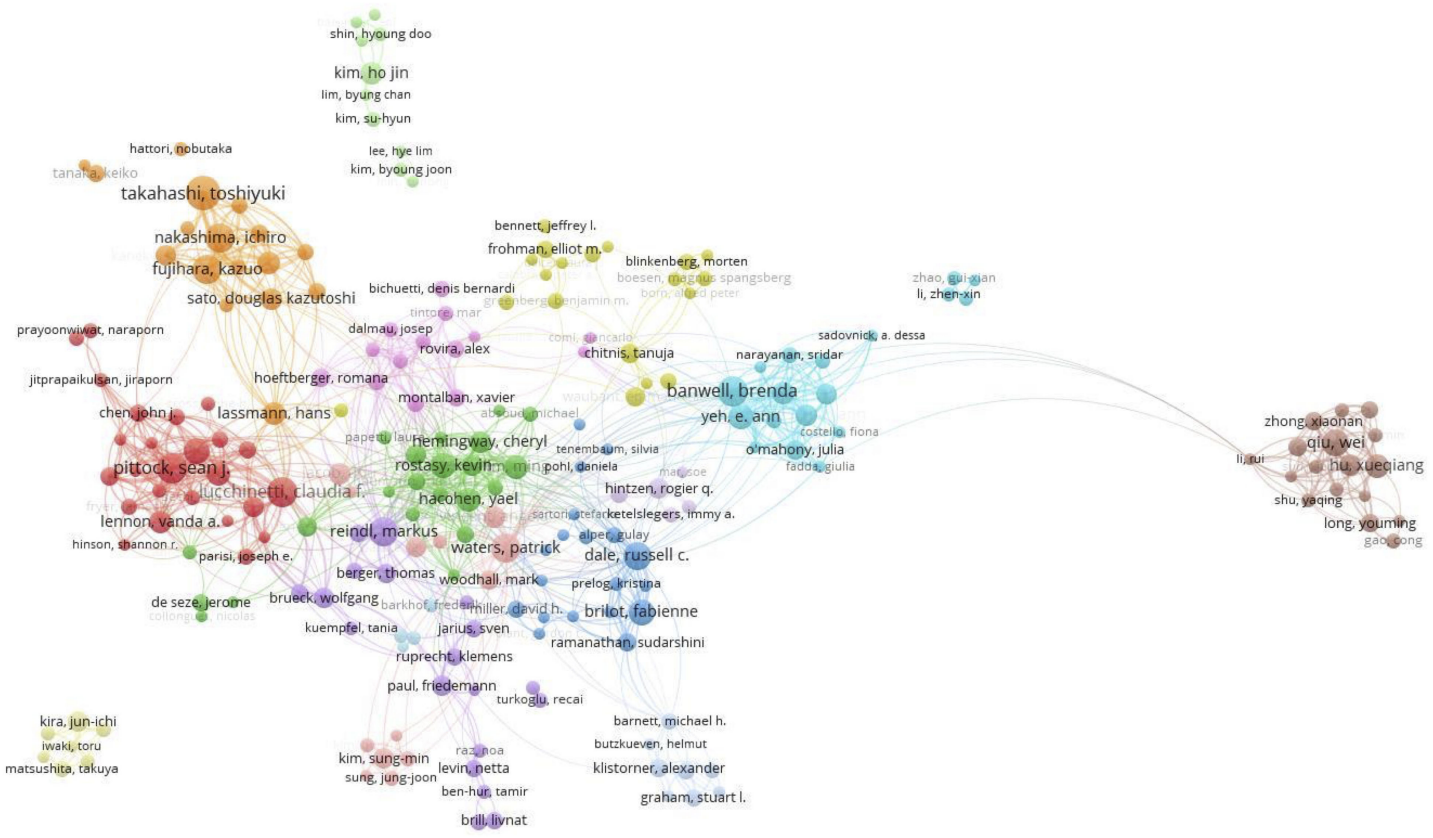
Cooperation network of authors.

**Figure 5** shows the author’s collaborative network relationship. The network reflects strong connections between authors from different countries. A total of 871 authors with a minimum of 5 documents were analyzed using VOSviewer. The top 5 authors with largest total link strength were as follows: Rostasy Kevin (total link strength =1975 times), WatersPatrick (total link strength = 1738 times), Reindl Markus (total link strength =1722 times), Lucchinetti Claudia F. (total link strength = 1373 times), and Wassmer Evangeline (total link strength = 1346 times).

### 3.5 Journals

**Table 3** lists the top 10 productive journals in terms of MOGAD related publications and their respective Journal Impact Factor (JIF). *Multiple Sclerosis and Related Disorders* (JIF=4.0, 2022) has the highest number of publications (144). There were 138 publications in *Multiple Sclerosis Journal* (IF =5.8, 2022), 95 publications in *Neurology* (IF=9.9, 2022), 83 publications in *Journal of Neuroimmunology* (IF=3.3, 2022), and 74 articles in *Journal of the Neurological Sciences* (IF= 4.4, 2022).

**Table 3:** Top 10 journals in the field of MOGAD related research.

Rank	Journals	IF (2022)	Article counts
1	MULTIPLE SCLEROSIS AND RELATED DISORDERS	4.0	144
2	MULTIPLE SCLEROSIS JOURNAL	5.8	138
3	NEUROLOGY	9.9	95
4	JOURNAL OF NEUROIMMUNOLOGY	3.3	83
5	JOURNAL OF THE NEUROLOGICAL SCIENCES	4.4	74
6	JOURNAL OF CHILD NEUROLOGY	1.9	52
7	JOURNAL OF NEUROLOGY	6.0	50
8	JOURNAL OF NEUROLOGY NEUROSURGERY AND PSYCHIATRY	11.0	42
9	BRAIN	14.5	39
10	FRONTIERS IN NEUROLOGY	3.4	39

**Figure 6:**
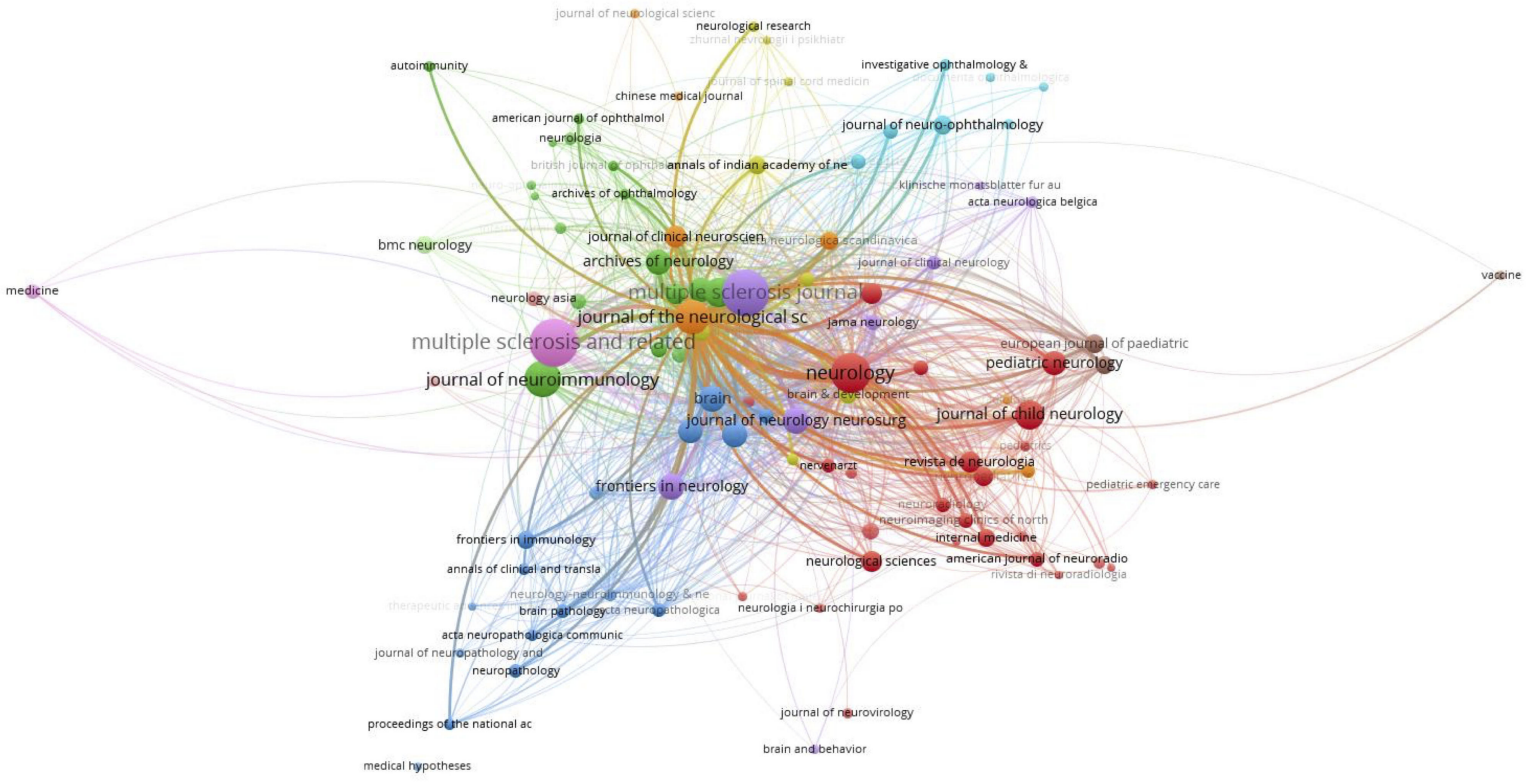
Co-occurrence network of journals.

**Figure 6** plots 98 journals in terms of the total link strength. The top 5 journals with best total link strength were as follows: Neurology (total link strength =1832 times), Multiple Sclerosis Journal (total link strength =1222 times), Brain (total link strength = 833 times), Multiple Sclerosis and Related Disorders (total link strength = 762 times), and Journal of Neurology, *Neurosurgery, and Psychiatry* (total link strength = 539 times).

**Figure 7:**
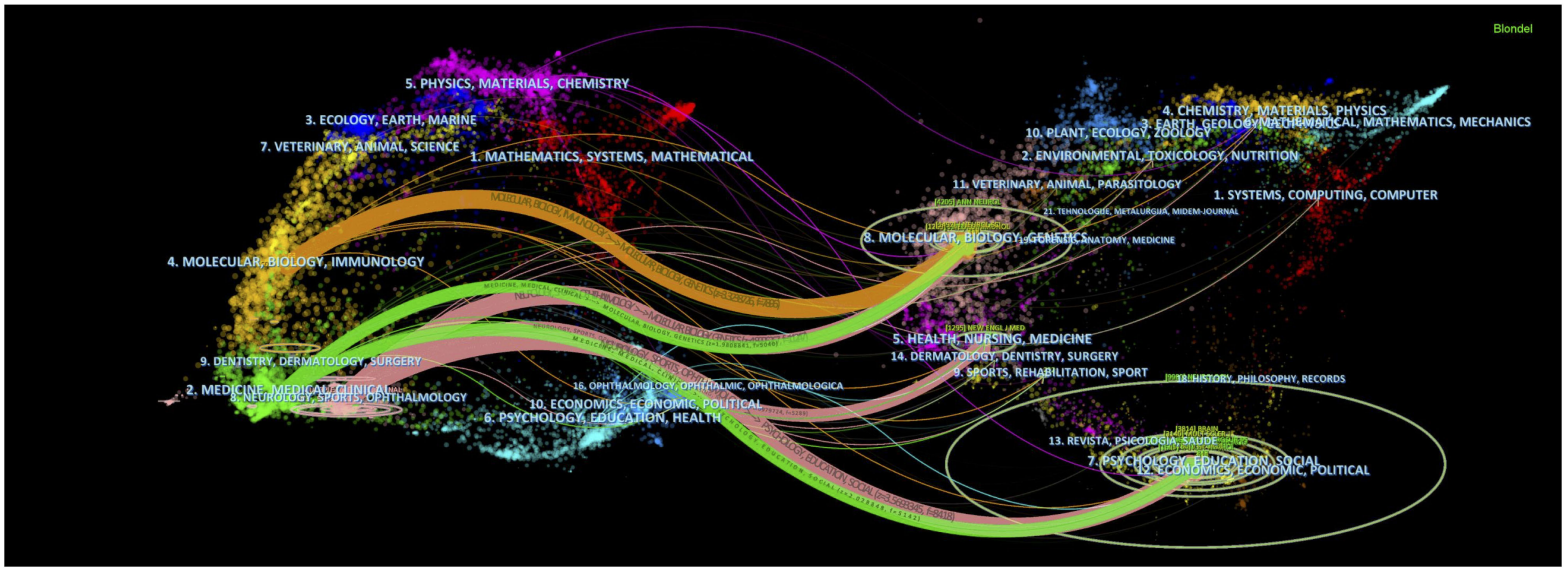
Journal overlay map related to MOGAD research.

The Journal Overlay Maps (**Figure 7**) is composed of a journal citing map (left) and a journal cited map (right) based on the clustering of discipline field. The co-citation links connecting the two maps shows the flow of disciplines. The horizontal axis of the ellipse represents the number of articles, and the vertical axis represents the number of authors. In the discipline field of Psychology, Education, Social, the number of authors and articles are more than others. There are 3 clusters (Medicine, Medical, Clinical/Molecular, Biology, Immunology/Neurology, Sports, Ophthalmology) in the journal citing map. And the discipline field has gradually developed to 3 clusters (Molecular, Biology, Genetics/Health, Nursing, Medicine/Psychology, Education, Social) shown in the journal cited map.

### 3.6 Citation and co-citation analysis

**Figure 8:**
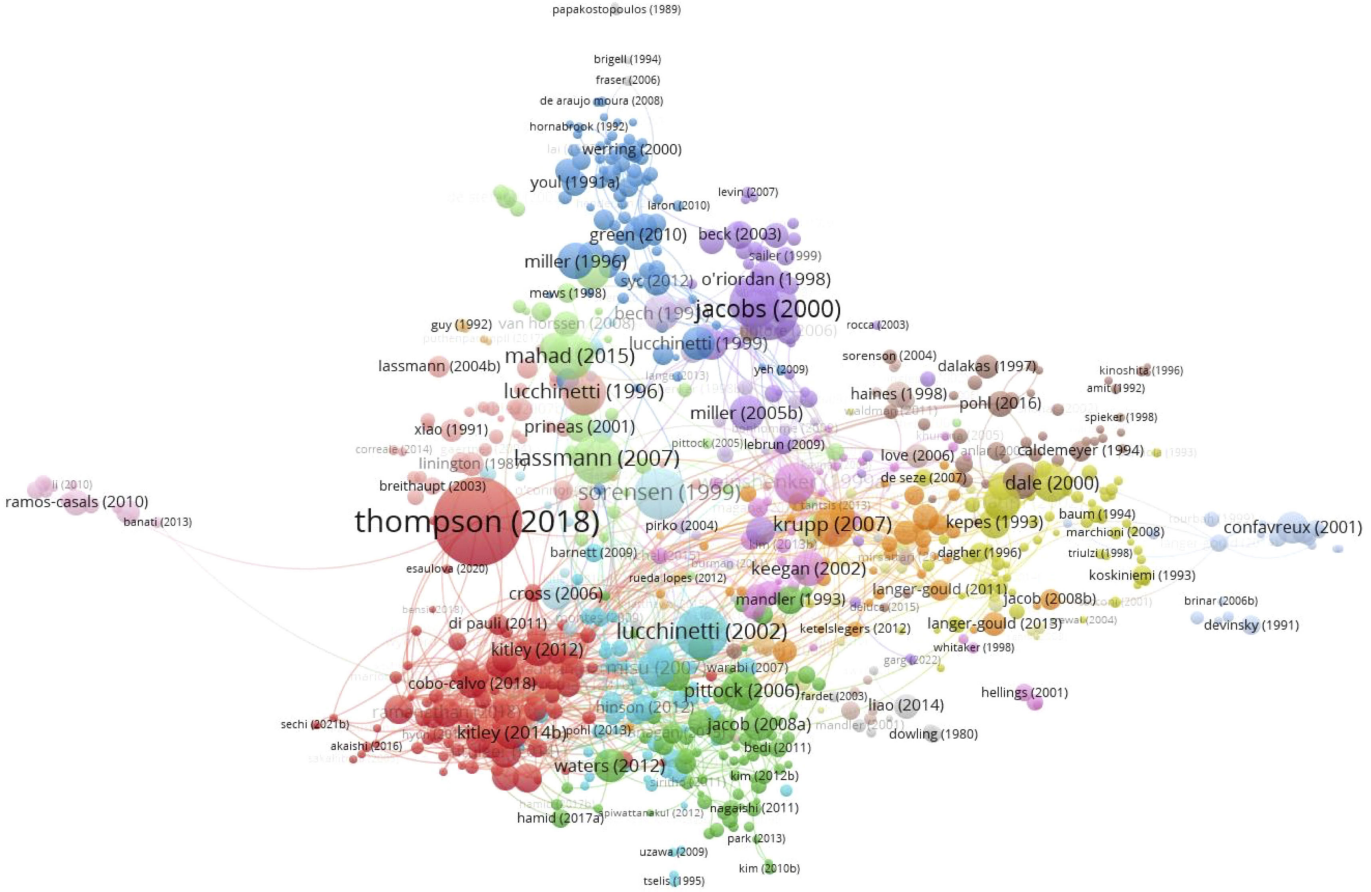
Citation network of articles related to MOGAD research.

A total of 841 articles in this field have more than 20 citations (**Figure 8**). The top 10 most cited documents are shown in **Table 4**. There were 2763 citations for “Diagnosis of multiple sclerosis: 2017 revisions of the McDonald criteria”, followed by 1146 citations for “Intramuscular interferon beta-1a therapy initiated during a first demyelinating event in multiple sclerosis”. The third most frequently cited article was “A role for humoral mechanisms in the pathogenesis of Devic’s neuromyelitisoptica” with 873 citations.

**Table 4:** Top 10 documents with the most citations in the field of MOGAD related research.

Rank	Title	Journal	JIF(2022)	Total citations
1	Diagnosis of multiple sclerosis: 2017 revisions of the McDonald criteria	LANCET NEUROLOGY	48.0	2763
2	Intramuscular interferon beta-1a therapy initiated during a first demyelinating event in multiple sclerosis.	NEW ENGLAND JOURNAL OF MEDICINE	158.5	1146
3	A role for humoral mechanisms in the pathogenesis of Devic’s neuromyelitisoptica	BRAIN	14.5	873
4	Expression of specific chemokines and chemokine receptors in the central nervous system of multiple sclerosis patients	JOURNAL OF CLINICAL INVESTIGATION	15.9	830
5	Effect of early interferon treatment on conversion to definite multiple sclerosis: a randomised study	LANCET	168.9	796
6	The immunopathology of multiple sclerosis: An overview	BRAIN PATHOLOGY	6.4	785
7	Progressive multiple sclerosis 1 Pathological mechanisms in progressive multiple sclerosis	LANCET NEUROLOGY	48.0	673
8	Distinct patterns of multiple sclerosis pathology indicates heterogeneity in pathogenesis	BRAIN PATHOLOGY	6.4	631
9	A randomized trial of plasma exchange in acute central nervous system inflammatory demyelinating disease	ANNALS OF NEUROLOGY	11.2	558
10	Consensus definitions proposed for pediatric multiple sclerosis and related disorders	NEUROLOGY	9.9	544

**Table 5** shows the top 10 most average cited documents per year. Compared to the total citations, the annual average citation index can better reflect the attention paid to articles, as the annual average citation takes into account the factor of publication time. This will avoid the issue of the popularity of articles published in recent years being overlooked because low total citations. There were 577 citations for “Diagnosis of multiple sclerosis: 2017 revisions of the McDonald criteria”, followed by 93 citations for “The spectrum of neuromyelitis optica”. The third most frequently cited article was “Progressive multiple sclerosis 1 Pathological mechanisms in progressive multiple sclerosis” with 82 citations.

**Table 5:** Top 10 documents with the most annual average citation in the field of MOGAD related research.

Rank	Title	Journal	JIF(2022)	Annual average citation
1	Diagnosis of multiple sclerosis: 2017 revisions of the McDonald criteria	LANCET NEUROLOGY	48.0	577
2	The spectrum of neuromyelitis optica	LANCET NEUROLOGY	48.0	93
3	Progressive multiple sclerosis 1 Pathological mechanisms in progressive multiple sclerosis	LANCET NEUROLOGY	48.0	82
4	Myelin oligodendrocyte glycoprotein antibodies in neurological disease	NEUROLOGY	9.9	65
5	International Pediatric Multiple Sclerosis Study Group criteria for pediatric multiple sclerosis and immune-mediated central nervous system demyelinating disorders: revisions to the 2007 definitions	NEUROLOGY	9.9	61
6	Clinical course, therapeutic responses and outcomes in relapsing MOG antibody-associated demyelination	JOURNAL OF NEUROLOGY NEUROSURGERY AND PSYCHIATRY	11.0	57
7	Clinical presentation and prognosis in MOG-antibody disease: a UK study	BRAIN	14.5	53
8	Clinical spectrum and prognostic value of CNS MOG autoimmunity in adults The MOGADOR study	NEUROLOGY	9.9	53
9	The immunopathology of multiple sclerosis: An overview	BRAIN PATHOLOGY	6.4	48
10	An updated histological classification system for multiple sclerosis lesions	ACTA NEUROPATHOLOGICA	12.7	48

### 3.7 Keywords and hotspots

In the co-occurrence analysis, a keyword was defined as the word that was used more than 5 times in titles or abstracts in all papers. 3295 keywords were extracted, of which 233 terms appeared more than 5 times. The top 10 keywords with the highest frequency in the field of MOGAD research are displayed in **Table 6**. “Multiple sclerosis” was the most frequent and strongest Link strength keyword. In addition, “neuromyelitis optica”, “optic neuritis”, “demyelination” and “acute disseminated encephalomyelitis” have stronger links among the keywords.

**Table 6:** Top 10 keywords with the highest frequency in the field of MOGAD related research.

Rank	Keyword	Occurrences	Total link strength	Average citations	Average publish year
1	multiple sclerosis	795	1899	24.2579	2011.8357
2	neuromyelitis optica	389	982	24.6375	2014.2403
3	optic neuritis	309	742	17.4401	2012.5578
4	acute disseminated encephalomyelitis	229	506	18.214	2011.0661
5	demyelination	206	519	26.5243	2011.2745
6	magnetic resonance imaging	128	353	23.4062	2009.7734
7	demyelinating disease	102	251	21.451	2013.1176
8	mri	96	250	25.9479	2011.2737
9	myelin oligodendrocyte glycoprotein	86	245	19.6744	2017.2791
10	neuromyelitis optica spectrum disorder	85	212	8.0941	2019.4286

**Figure 9:**
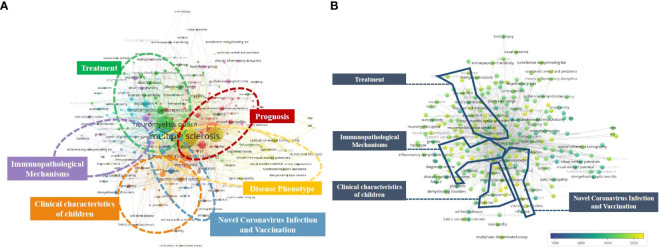
**(A)** Keyword co-occurrence network diagram of MOGAD. **(B)** Keyword co-occurrence average years network diagram of MOGAD.

In the co-occurrence analysis, a keyword was defined as the word that was used more than 5 times in titles or abstracts in all papers. The selected keywords were analyzed via VOSviewer. **Figure 9A** shows six clusters classified from 233 identified keywords. Cluster 1: Disease Phenotype (yellow); Cluster 2: Treatment (green); Cluster 3: Novel Coronavirus Infection and Vaccination (dark blue); Cluster 4: Immunopathological Mechanisms (Purple); Cluster 5: Clinical characteristics of children (orange); Cluster 6: Prognosis (red). In the “Disease Phenotype”cluster, the frequently used keywords were demyelination, ADEM, and NOMSD. In the “Treatment” cluster, the primary keywords were rituximab, corticosteroids, and immunoadsorption. In the “Novel Coronavirus Infection and Vaccination” cluster, the frequently used keywords werecovid-19, sars-cov-2, and vaccine. In the “Immunopathological Mechanisms” cluster, the dominantly used keywords were aquaporin 4, auto-antibodies, and autoimmunity. In the “Clinical characteristics of children” cluster, the main keywords used were children, acute disseminated encephalomyelitis, and epidemiology. Prognosis, outcome, acute transverse myelitis were the most commonly used keywords in the “Prognosis” cluster. The results exhibited the six most prominent directions of MOGAD-related research.

In **Figure 9B**, the VOSviewer colored all keywords based on the average publication year they appeared in the MOGAD papers. Specifically, the yellow color indicates a more recent appearance (new words), while blue color indicates that the keywords appeared relatively early (old words).

The research trends of most studies in the six clusters are related to the novel coronavirus and vaccine (cluster 3), immunopathological mechanism (cluster 4), clinical characteristics of children (cluster 5), and treatment (cluster 2), suggesting that future research hotspots may be related to the novel coronavirus, pathogenesis, and special population.

## 4 Discussion

### 4.1 Trends in the development of MOGAD-related research

Recent decade shave witnessed significant research on MOGAD, resulting in noteworthy advances in the understanding of disease phenotypes and their classification. However, there is a lack of international consensus regarding the diagnostic criteria for MOGAD. Moreover, there are several gaps in our understanding of the pathogenesis, treatment, and prognosis of MOGAD. At present, there is no bibliometric analysis on MOGAD. Therefore, the present study provides useful reference for relevant researchers to identify new research ideas and directions. This study provides an overview of the trends in the emergence of MOGAD. Notably, there has been a substantial rise in the annual publication count, particularly after 1991. Furthermore, the relative research interest (RRI) has exhibited a slight upward trajectory in recent years, signifying a growing popularity of this research domain. Approximately 72 countries have published research papers pertaining to MOGAD. Notably, the largest number of articles was published in the United States. Our study shows that the United States has high research quality and great academic influence in the field of MOGAD research, and has made the greatest contribution to publications. It also reflects that the United States has the most outstanding researchers and institutions in the world, and is a highly productive and leading country in the field of MOGAD. We found that while the Netherlands only ranks 15th in the number of publications, it has made significant progress in the average frequency of citations per article, ranking third. At the opposite end of the spectrum is China, which, although ranked second in terms of total number of publications, performs relatively poorly in terms of total and average number of citations. Therefore, China needs to investigate and resolve the contradiction between the quantity and quality of research published by Chinese researchers in order to further improve the quality of MOGAD related research. The Mayo Clinic USA, University College London, and the University of Sydney have made significant contributions to MOGAD related research. Notably, approximately 60% of research institutions are located in the top five countries by volume, indicating that leading research institutions play a pivotal role in enhancing a country’s academic research ranking. Our study shows that future research on MOGAD needs to be conducted through further cooperation and exchanges with leading countries to guide researchers to publish higher quality papers.

### 4.2 Status and quality of authors, journals, and studies

The Japanese authors with the highest ranking and most publications hold a critical position in the field of MOGAD, indicating Japan’s significant contribution. These early participants have likely pioneered new advancements in the field of MOGAD. Additionally, based on collaborative analysis, the research relationships between authors from different countries appear to be relatively dispersed, indicating a lack of academic connections and communication among them. Therefore, it is imperative for authors from diverse countries and institutions to enhance collaboration in order to collectively advance research on MOGAD. Almost all the studies related to MOGAD have been published in journals in the field of neurology. The top 5 journals with the highest combined link strength are *Neurology, Journal of Multiple Sclerosis, Brain, Multiple Sclerosis and Related Disorders, and Journal of Neurology Neurosurgery and Psychiatry*, which will be the journals of choice for researchers to publish high-quality research in the future. According to the results of co-citation and total citation analysis, *Lancet Neurology* has made significant contributions in the field of MOGAD research. Although the number of publications is small, the quality of publications in the fields of molecular biology, immunology and pharmacology is prioritized.

### 4.3 Research hotspots and frontiers

The co-occurrence analysis of keywords and bursts provides insights into the development trends and hotspots of MOGAD related research. “Multiple sclerosis” was the most frequently cited burst keyword, indicating its significance in the early stages of research on MOGAD. For instance, in the 1990s, Xiao BG et al. identified MOG as a potential target for antibody-mediated immune attack in experimental autoimmune encephalomyelitis (EAE), which has been extensively used as the model of MS. This demonstrated the significance of anti-MOG antibodies in MS CSF. The cluster analysis performed in our study not only reflects the primary research trends in MOGAD but also provide insights into future research directions.

#### 4.3.1 Phenotypes

The last 15 years have witnessed a significant shift in the global concept of inflammatory demyelinating disorders of the CNS, with the identification of distinct autoantibody-associated disorders such as aquaporin 4-IgG-positive neuromyelitisoptica spectrum disorder and MOGAD, which are distinct from MS (5, 37–39). MOGAD is a newly identified autoimmune disorder manifesting as CNS demyelination in both adults and children. It can present with various clinical phenotypes, including ON, myelitis, encephalitis, ADEM, NMOSD, or any combination of these entities. The frequency of MOGAD varies depending on the phenotype in both adults and children. In a single-center retrospective study, serum anti-MOG antibodies were detected in 60% (12 out of 20) of adult patients with acute disseminated encephalomyelitis (ADEM) either at onset or during follow-up (17). In a population-based Danish study, anti-MOG antibodies were detected in 4% (2/51) of adults with initial ON (26). Additionally, in two retrospective studies of individuals with anti-APQ4 antibody seronegative longitudinal extensive TM, 16-23% of cases tested positive for anti-MOG antibodies (40, 41). MOG antibodies are detectable in up to 64% of children with ADEM and in nearly all children who relapse after acute disseminated myelitis (including multistage ADEM or acute NMOSD) (13, 42, 43). However, MOG antibodies were detected in 33-43% of pediatric patients with ON and only in 3 (6%) out of 50 children with myelitis (22). As the understanding of MOGAD continues to evolve, there is a constant update on its clinical phenotype, which has become a research hotspot.

#### 4.3.2 Treatment

In 1999, a groundbreaking randomized controlled double-blind trial demonstrated the effectiveness of plasma exchange in patients with severe demyelinating episodes who did not respond to intravenous glucocorticoids (44). Observational studies have shown that a brief course of intravenous steroids can achieve significant symptom relief in MOGAD (18, 19, 45, 46). In patients who do not respond to intravenous glucocorticoids or those with severe disease, plasma exchange, immunoadsorption therapy, intravenous immune globulin, or a combination of plasma exchange followed by intravenous immune globulin may be necessary (47). Patients with a monophasic course of MOGAD account for 40-50% of cases, and the majority exhibit a favorable prognosis. Consequently, there is no clear consensus on the need for long-term immunosuppressive therapy. In general, preventative immunotherapy should be considered for patients who experience two or more relapses or severe functional disability following their initial episode (11, 48, 49).The majority of current literature is based on real-world clinical data from MOGAD patients, and there is a dearth of clinical trials pertaining to MOGAD treatment as well as related studies examining the risk factors for recurrence and predictors of long-term efficacy. These areas are poised to emerge as key areas of focus in future MOGAD research.

#### 4.3.3 COVID-19 and vaccination

Following the emergence of the 2019 novel coronavirus, there has been a surge in literature pertaining to COVID-19, SARS-CoV-2, and vaccines. In 2021, Japanese scholar Kenji Kashiwagi reported the first case of acute ON associated with MOG antibody potentially induced by COVID-19 in adults (50). With the increasing number of case reports, large-scale studies, and reviews on COVID-19 in patients with NMOSD and MOGAD, there has been a gradual increase in knowledge regarding this topic (51–54). Aksel Siva et al. (2022) conducted a retrospective study of 63 COVID-19 patients with NMOSD and MOGAD from 25 centers. They found that advanced age, high disability rate, and comorbidities were risk factors for severe COVID-19 in NMOSD and MOGAD patients (55).Studies have shown that approximately 20% of MOGAD cases manifest initially following COVID-19 infection or vaccination (6, 56–58). The literature provides a comprehensive review of the epidemiological, clinical, imaging, electrophysiological, and laboratory characteristics as well as treatment outcomes for patients with SARS-CoV-2 and COVID-19 vaccine-associated MOGAD. Such patients exhibit severe symptoms at onset and typically require escalated immunotherapy while maintaining long-term persistence of MOG-IgG (59). Previous studies have indicated that approximately 30% of patients with MOGAD exhibit a prodromal history of infection or vaccination (60). However, the overall risk for developing MOGAD or experiencing a relapse after SARS-CoV-2 infection/vaccination appears to be exceedingly low (61). These findings suggest that certain novel environments associated with an elevated risk for neuroautoimmune disease development may also be linked to the emergence of MOGAD.

#### 4.3.4 Immunopathological mechanisms

The trigger for the production of anti-MOG antibodies remains unknown, but is likely attributable to the induction of autoimmunity in the peripheral immune system. Although infection-induced autoimmunity may serve as a trigger, disease-specific pathogens have yet to be identified. Potential mechanisms of post-infection autoimmunity include molecular mimicry, bystander activation, epitope spreading, B-cell receptor-mediated antigen co-capture, and polyclonal activation of B cells (62–64). The presence of anti-MOG antibodies and plasma cells can also augment the activation of cognate MOG-specific CD4+ T cells or myelin basic protein-specific T effector cells and macrophages in the CNS (65). Kaneko K et al. found elevated levels of proinflammatory cytokines (IL-6, IL-17, G-CSF, and TNFα), as well as B-cell cytokines and chemokines (BAFF, APRIL, CXCL13, and CCL19) in the CSF of patients with MOGAD compared to that in healthy controls (62). It has been proposed that anti-MOG antibodies can directly trigger the classical pathway of the complement cascade, leading to demyelination. Both children and adults with MOGAD exhibit a significant increase in proteins indicating systemic activation of the classical and alternative complement pathways, which may result in CNS damage caused by activated complement proteins (66). Due to the gaps in our understanding of the pathogenesis of MOGAD, research on immunopathological mechanisms is currently a key research focus.

#### 4.3.5 Clinical characteristics of the pediatric population

The seropositivity rate of MOG-IgG in pediatric patients is higher than that in adults (7), with approximately 34% of children diagnosed with acquired demyelinating diseases testing positive for MOG antibodies. There is no significant seasonal variation observed in the onset or recurrence of MOG-IgG related disorders (8).No significant sex-based differences have been noted (male-to-female ratio 1:1.3) (9). MOGAD is characterized by age-dependent phenotypic expression (67), seizure severity, and recovery outcomes (60).The primary clinical phenotypes observed in children with MOGAD are ADEM (53%), ON (40%), and TM (18%) (2). Children with ADEM typically exhibit multifocal and asymmetric white matter abnormalities on MRI, while those with leukodystrophy tend to present with fused, bilateral, and essentially symmetric white matter abnormalities on MRI. These abnormalities are more prevalent in young children (mean onset age: 3.7 years), and their prognosis is worse than that of other patients, possibly due to the immaturity of CNS development (68). Older children are more prone to present with ON and/or TM as well as brainstem symptoms (22, 68). The coexistence of MOGAD and other autoantibodies has been reported, with double-positive patients exhibiting greater overlap in clinical manifestations with NMDAR encephalitis and greater similarity in MRI changes with MOGAD (69, 70). While children have a relatively low risk of recurrence, the majority of other patients experience a monophasic course (22).Less than10% of relapsing children (typically very young) exhibit a leukodystrophic phenotype characterized by large confluent hyper-enhancing lesions on MRI and progressive brain atrophy leading to permanent cognitive and motor disability (68).

#### 4.3.6 Prognosis

Despite favorable clinical outcomes in the majority of MOGAD patients, recurrence rates remain high and a subset of patients may experience residual disabilities including visual impairment, motor dysfunction, cognitive deficits, sensory disturbances, bladder dysfunction, and epilepsy (19). Patients with the TM phenotype are more likely to experience physical dysfunction, whereas those with the ON phenotype tend to exhibit less disability (19, 71). Relapse is defined as the onset of new symptoms at least three months after disease onset, with or without steroid treatment (24, 25). Numerous studies have reported that patients exhibiting certain factors are more prone to experiencing a recurrent course, including clinical phenotype of ON, persistent high levels of MOG antibodies, MRI manifestations of cortical encephalitis and leukodystrophy, as well as concomitant presence of other autoantibodies such as anti-NMDAR antibody (63, 68, 69). A study revealed decreased brain volume in adults with MOGAD compared to healthy individuals (72). Furthermore, patients with recurrent episodes exhibited significant reductions in total brain volume, deep gray matter, cerebellum, and hippocampus when compared to those with a monophasic course (72). Several studies have suggested that the neutrophil-to-lymphocyte ratio can serve as a potential biomarker for predicting recurrence in MOGAD. This ratio only increases during the clinical onset of MOGAD and remains stable during remission, indicating its correlation with disease activity. These findings may aid in more accurate recurrence prediction and facilitate improved treatment decisions (73).

### 4.4 Future research trends

The aforementioned analysis provides insights on the likely future trends and potential impacts of diseases related to MOG antibodies. According to a review published in *Nature Reviews* in 2018, future studies will focus on the mechanisms of MOGAD tolerance and age-dependent mechanisms of disease recurrence, and the genetic factors associated with MOGAD need to be identified. A review published in *The Lancet* in 2021 stated that future research should be more important to understand the mechanism behind the development of MOG autoimmune response, and there is an urgent need to identify disease-specific biomarkers of outcome and treatment response, which may pave the way for antigen-specific immunotherapy (5, 6). Similar to our research trend, with the advancement of medicine, the focus of research has shifted from clinical phenotype/characteristics to special populations, biomarkers, and molecular biological mechanisms. This shift will have a significant impact on future research. The patients exhibiting diverse phenotypes and possessing anti-MOG antibodies may harbor distinct underlying pathobiology driving their disease. In the future, advancements in *in-vivo* and *in-vitro* models, such as human-derived oligodendrocyte cultures, rodent models expressing humanized MOG, or animal models featuring MOG proteins with a higher homology to human MOG than rodents, will offer an enhanced foundation for investigating the pathogenic mechanisms. The identification of markers indicating disease outcome holds paramount significance for clinicians to propose optimal therapy and management strategies at disease onset. For future research, the formulation of a sophisticated analytical approach is crucial, particularly considering that only antibodies capable of recognizing properly folded MOG protein exhibit pathogenicity. The identification of new biomarkers may prove instrumental in monitoring disease activity in MOGAD. It is imperative to establish treatment protocols for MOGAD in the coming years. Investigating antigen-specific CD4+ T-cells and B-cells will be pivotal in gaining a deeper understanding of the mechanisms underlying autoimmune responses to MOG and will pave the way for antigen-specific immune therapies. Conducting multicenter international studies will not only expand our current knowledge but also enable us to evaluate initial therapy options as well as intensified therapeutic approaches.

### 4.5 Strength and limitations

The significant advantage of our study lies in the extensive analysis of global publications on MOGAD from the perspective of scientific literature. Bibliometric analysis methods in this study are necessary and valuable, which can quantitatively reflect research status and practical applications simultaneously. It is important to be aware of the limitations of the work. The first issue concerns the search strategy. We use the database of Web of Science Core Collection (WoSCC) for analysis, which it is the most commonly used bibliometric analysis database. Because the format of literature data in WoSCC is very standardized and has a very complete citation network relationship. Moreover, the existing knowledge map software support the extraction and meta-analysis of multi-database records from WoSCC very much. The realistic dilemma is that the document included in one database may not be comprehensive. Using the other citation databases such as Scopus or SciFinder somewhat different results may be found. The second issue concerns the bibliometrics, which has been debated by some researchers. In our study, quantitative metrics such as the number of publications and the number of citations (Total citations and Annual average citation) were used to reflect the co-authorship, co-occurrence, and co-citation. Some researchers like Garfield (74) and Lynch (75) think these metrics above should not be understood as quality measures or endorsements of the research outputs, and high citation rate does not imply correctness or impact beyond the academic context. So, developing more objective quantitative metrics is one of the important goals in the future. In addition, since the research included in this paper is not comprehensive enough on the research about the identification of markers of the disease status and outcome and the establishment of *in vivo* and *in vitro* models of MOGAD. Finally, prospective randomized controlled trials with large sample sizes should be conducted to establish the most optimal way of care and treatment strategy.

## 5 Conclusion

In conclusion, this study represents the first comprehensive bibliometric analysis of global research related to MOGAD since 1947. The systematic summary of global publishing trends identifies the leading authors, institutions, and journals in the field. Furthermore, the utilization of key words and co-citation cluster analysis identifies the main research avenues, primarily encompassing “phenotypes”, “treatment”, “COVID-19 and vaccination”, “immunopathological mechanism”, “The clinical characteristics of the pediatric population” and “prognosis”. It is anticipated that future collaborations among authors, institutions, and countries will expedite advancements in the study of MOGAD.

## Data availability statement

The original contributions presented in the study are included in the article/supplementary material. Further inquiries can be directed to the corresponding author.

## Author contributions

SZ: Conceptualization, Data curation, Writing – original draft. YW: Conceptualization, Data curation, Writing – original draft. JG: Methodology, Software, Writing – review & editing. XL: Formal analysis, Methodology, Writing – review & editing. LH: Formal analysis, Funding acquisition, Supervision, Visualization, Writing – review & editing.
